# An Anatomical Study on Canine Cadavers Investigating the Caudolateral Approach Involving the Elevation of the Anconeus Muscle and Splitting of the Triceps Brachii Muscle for the Potential Treatment of T-Y Humeral Fractures

**DOI:** 10.3390/ani16010110

**Published:** 2025-12-30

**Authors:** Piotr Trębacz, Jan Frymus, Michał Czopowicz, Anna Barteczko, Mateusz Pawlik, Aleksandra Kurkowska

**Affiliations:** 1 Department of Surgery and Anesthesiology of Small Animals, Institute of Veterinary Medicine, Warsaw University of Life Sciences-SGGW, Nowoursynowska 159c, 02-776 Warsaw, Poland; 2 Division of Veterinary Epidemiology and Economics, Institute of Veterinary Medicine, Warsaw University of Life Sciences-SGGW, Nowoursynowska 159c, 02-776 Warsaw, Poland; michal_czopowicz@sggw.edu.pl; 3 CABIOMEDE Ltd., K. Olszewskiego 6, 25-663 Kielce, Poland; anna.barteczko@cabiomede.com; 4 Department of Biomaterials and Medical Devices Engineering, Faculty of Biomedical Engineering, Silesian University of Technology, 41-800 Zabrze, Poland; mateusz.pawlik@cabiomede.com (M.P.); aleksandra.kurkowska@cabiomede.com (A.K.)

**Keywords:** dog, humeral condyle, humeral fracture, olecranon

## Abstract

Due to the complex anatomical structure of the distal humerus, elbow joint, and the soft tissue mantle (the triceps brachii muscle, large nerves, and vessels), fractures of the distal humerus and humeral condyle are difficult to treat. In most cases, strong instrumentation is needed to stabilize the fractures. To improve exposure of the distal humerus and humeral condyle, we proposed a caudolateral approach that involves elevating the anconeus muscle and splitting the triceps brachii. This study presents the results of using this approach in 16 canine cadavers. After exposing the distal humerus and maximally flexing the elbow joint, photographs were taken of the condyle from the same distance before and after olecranon osteotomy. The visible surface area of the articular cartilage was then calculated in square pixels after calibrating the photographs. It was possible to reach the distal and middle humerus in all cases. The only vital structure that could be easily identified and protected in all cadavers was the radial nerve. The visible area of the articular surface of the humeral condyle increased after olecranon osteotomy. The A_0_ (visible area before osteotomy) was significantly smaller than the A_1_ (visible area after osteotomy) in all dogs (*p* < 0.001). The ratio of A_0_ to A_1_ ranged from 57% to 67% in 15 dogs (median: 64%, interquartile range (IQR): 61–66%), with a very high value of 85% observed in one dog. This experiment used cadavers with intact elbows. This could limit the study’s findings because the effectiveness of the proposed access in reducing T-Y fractures was not assessed. The caudolateral approach is a valuable alternative to other methods for treating T-Y humeral fractures in dogs. Olecranon osteotomy widens access to the condyle. Further studies are needed to evaluate the necessity of olecranon osteotomy in clinical cases.

## Simple Summary

Humeral condylar and epicondylar fractures (T-Y fractures), are one of the most challenging orthopedic injuries to treat in dogs. Despite advances in internal fixation techniques, complications following repairs to these fractures often occur. It is widely recognized that achieving anatomical reduction in intra-articular fractures is important for restoring joint function. Due to the anatomical complexity of the distal humerus and elbow joint, it is often difficult to expose all lines of a T-Y fracture appropriately. Currently, the combined lateral and medial approach is the preferred method for treating distal humeral fractures in small animals. This approach provides good exposure of the lateral and medial epicondyles and the humeral diaphysis. However, access to the articular surface of the condyle is limited. In this anatomical cadaveric study, we present the results of using the caudolateral approach, which involves elevating the anconeus muscle and splitting the triceps brachii. We find this approach useful because it provides good access to the condyle, the epicondyles, and the humeral shaft. It can also help surgeons see fracture lines more clearly during repair. Appropriate reduction and instrumentation of T-Y fractures are crucial for restoring elbow joint function.

## 1. Introduction

Around half of all humeral fractures in dogs are fractures of the humeral condyle [1]. In humans, the T-Y fracture mechanism involves the ulna acting as a wedge to split the humeral condyle [2]. Such fractures can be assumed to occur in a similar way in small animals. Most fractures of the humeral condyle in dogs result from falling from a height. Upon impact, forces travelling from the distal end to the proximal end of the front limb can cause the elbow to flex until the lower part of the ulna touches the ground. In this position, the ulnar notch acts as a wedge, separating the trochlea and capitulum [1].

The T-Y fractures are challenging to treat due to the intricate anatomy of the distal humerus. A thick soft tissue envelope surrounds the distal humerus, comprising a large and strong triceps brachii muscle, as well as large nerves and blood vessels, which compromise the surgical approach to such fractures. Furthermore, distal humeral fractures may be comminuted, with small intra-articular fragments that are difficult to reposition and fixation. All of these factors increase the difficulty of the surgical procedure, thereby increasing the risk of postoperative complications and elbow stiffness [3].

The importance of achieving anatomical reduction and stable fixation in intraarticular fractures, if joint function is to be restored, is widely recognized. To achieve this, the fracture fragments must be accessible. The distal humerus consists of three columns that form a triangle: the medial, lateral, and transverse intercondylar columns. The stability of the bone depends on the integrity of this triangular structure [4,5,6]. The use of a Y-shaped bone plate or two plates inserted in a V configuration, alongside a transcondylar screw, fulfills these conditions. Use of bone plates requires a wide approach to the bone shaft. During the surgery, the intra-articular fracture can be reduced first. This is particularly true when the supracondylar fractures are comminuted. In some cases, such as those involving simple supracondylar fractures, it is preferable to reduce and stabilize the medial supracondylar fracture first, followed by the intra-articular and lateral supracondylar fractures [7,8,9,10,11,12,13].

Despite the limited access to the articular surface of the distal humerus (the triceps brachii caudally, the biceps brachii cranially, the radial nerve laterally, and the brachial artery and vein, as well as the median and ulnar nerves, medially), the combined lateral and medial approach is now a popular option for treating T-Y fractures in small animal surgery [4,5]. This is because the combination provides good access to the supracondylar region and humeral shaft, enabling robust instrumentation of fractures [7,9,13]. Although other approaches to the distal humerus offer better exposure of the distal articulation of the humerus, such as the transolecranon approach (olecranon osteotomy) [10], triceps tenotomy [11], and proximal ulnar diaphyseal osteotomy [12], they are not widely used due to the potential complications. For example, dysfunction of the extensor mechanism and destabilization of the osteotomy site. An approach that combines good access to the distal humerus, including the articular surface, epicondyles, and the bone shaft, is needed in canine surgery. Knowledge and development of new surgical approaches are important for surgeons. A correct choice of the surgical approach and the ability to use it appropriately in practice are basic prerequisites for a successful operation. Over the past 130 years, surgical approaches in human bone surgery have undergone considerable evolution. In 1892, Theodor Kocher became the first surgeon to emphasize that descriptions of surgical approaches should be an integral component of surgical textbooks on operative techniques [13]. Veterinary surgeons also began to recognize the need to include such descriptions in textbooks and papers [14,15,16].

Several posterior (caudal) approaches have been described in humans to provide access to the humeral condyle, epicondylar region, and shaft for the fixation of distal humeral fractures. All of these methods are variations in the three principal approaches, including, the transolecranon approach, triceps reflection, and triceps splitting [8,17,18]. The transolecranon approach can be used as a sole method or in combination with the triceps splitting or reflecting. No clinical advantage has been demonstrated for either approach over the other [19]. The olecranon osteotomy is commonly employed in complex distal humerus fractures due to its ability to provide a wide, clear, and detailed visualization of the articular surface of the distal humerus [17,20]. However, as with the transolecranon approach in small animals [21], complications in humans also include nonunion, symptomatic malunion, and implant loosening [22,23,24]

In humans, the triceps splitting is a surgical procedure that is widely regarded as safe and effective. The two most popular approaches are the Campbell and Van Gordner methods. In the Campbell approach, the triceps is split longitudinally through the midline of the triceps aponeurosis down to the bone. Van Gordner provided an alternative approach, raising a distal tongue of the triceps fascia in an inverted-V pattern before splitting the triceps longitudinally, leaving an extensor mechanism aponeurosis cuff intact distally to allow secure repair of the extensor mechanism. The remaining triceps muscle is then split longitudinally, as described by Campbell [25,26]. Triceps splitting between the long and lateral heads is also often used in humans to treat distal humeral fractures [18]. Ganta et al. [18] compared triceps splitting between the long and lateral heads, as well as triceps sparing, in humans. Their study suggests that the triceps-sparing approach does not offer any significant functional advantages over the triceps-splitting approach for treating extra-articular distal humerus fractures. Despite the theoretical benefits of preserving triceps integrity, there were no statistically significant differences in functional outcomes between the two groups at any time point. Additionally, both groups displayed comparable times to radiographic healing, with shorter operative times in the triceps-splitting group. However, these differences were not statistically significant. Similarly, the incidence of radial nerve palsy was low in both groups, suggesting that the approach is unlikely to affect this outcome. Overall, these findings suggest that the triceps-sparing technique, despite its aim to minimize muscle disruption, does not yield superior patient-reported outcomes or faster fracture healing.

**Figure 1:**
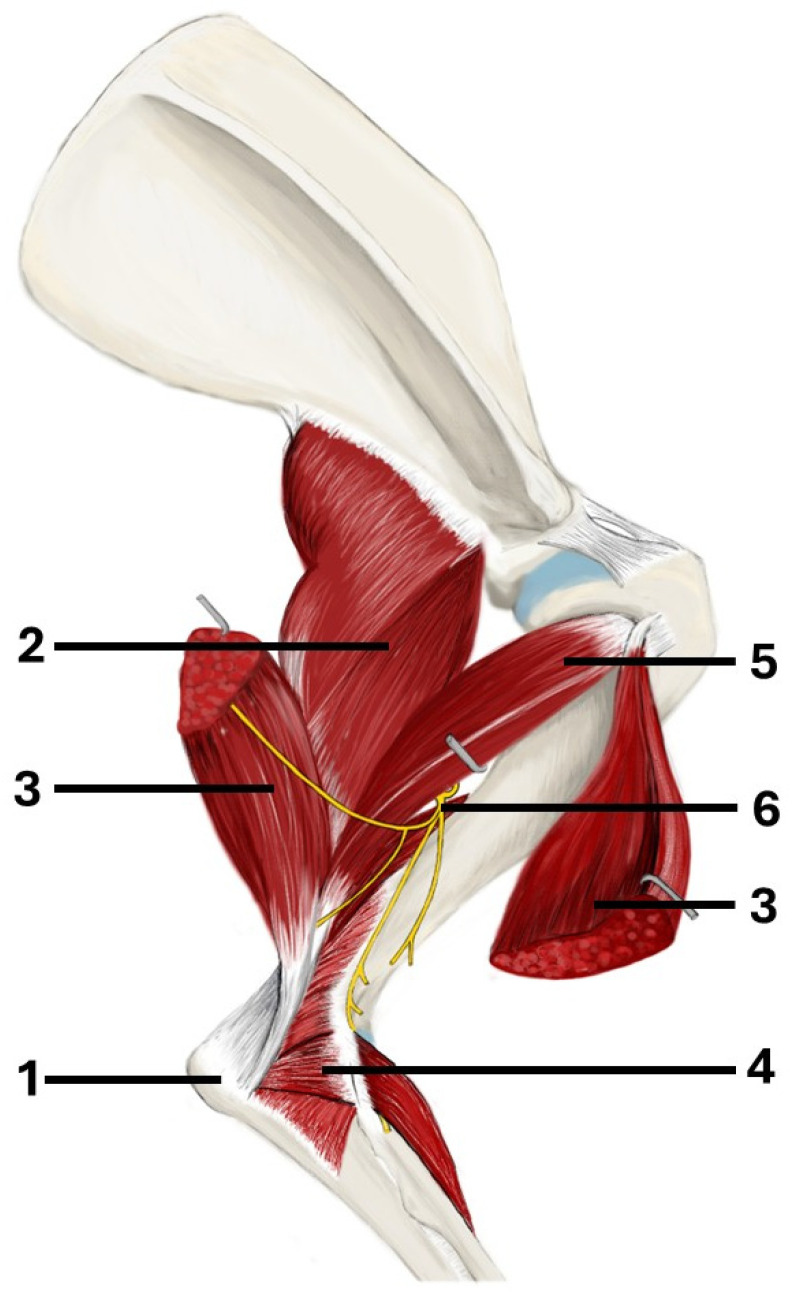
The anatomy of the canine triceps brachii and anconeus muscle (right forelimb): 1—olecranon, 2—long head of triceps brachii, 3—lateral head of triceps brachii, 4—anconeus muscle, 5—accessory head of triceps brachii, 6—the radial nerve.

The aim of this study was to develop a latero-caudal approach to the humeral condyle, supracondylar region, and bone shaft, based on approaches used in humans. Unlike in humans, the canine triceps brachii comprises four heads: the long head, the lateral head, the medial head, and the accessory head [27] (Figure 1). The additional head could potentially hinder triceps splitting in dogs. The proposed approach involves combining techniques to elevate the anconeal muscle and split the triceps brachii into the long and lateral heads, as well as the lateral and accessory heads. Additionally, an olecranon osteotomy was performed to ascertain whether this would improve access to the distal humeral articular surface.

## 2. Materials and Methods

The study included the cadavers of 16 adult dogs: nine males and seven females. Fourteen of the dogs were mongrels, one was a Boxer, and one was a Beagle. None of the dogs exhibited any visible pathologies affecting their front limbs. The dogs’ body weight ranged from 13 to 26 kg, with a median (IQR) of 21 (17–24) kg, and did not differ significantly between sexes (*p* = 0.958). Dogs were euthanized for reasons unrelated to this study. Immediately after euthanasia, cadavers were frozen at −20 °C. A day before the experiment, they were removed from the freezer and allowed to thaw at room temperature until all limbs could be freely flexed and extended. According to Polish legislation, ethics approval was not required (Act of the Polish Parliament of 15 January 2015 on the Protection of Animals Used for Scientific or Educational Purposes, Journal of Laws 2015, item 266) [28].

**Figure 2:**
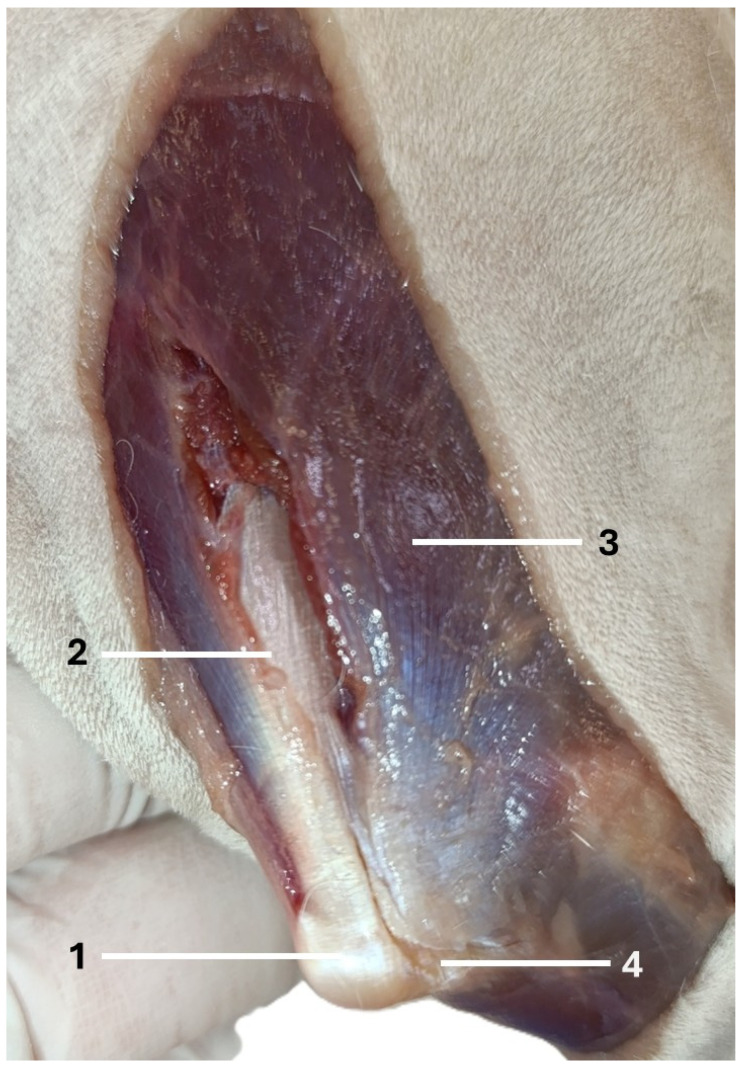
The first step in performing the caudo-lateral approach to the distal humerus (right forelimb): 1—olecranon, 2—long head of the triceps brachii, 3—lateral head of the triceps brachii, 4—anconeus muscle.

**Figure 3:**
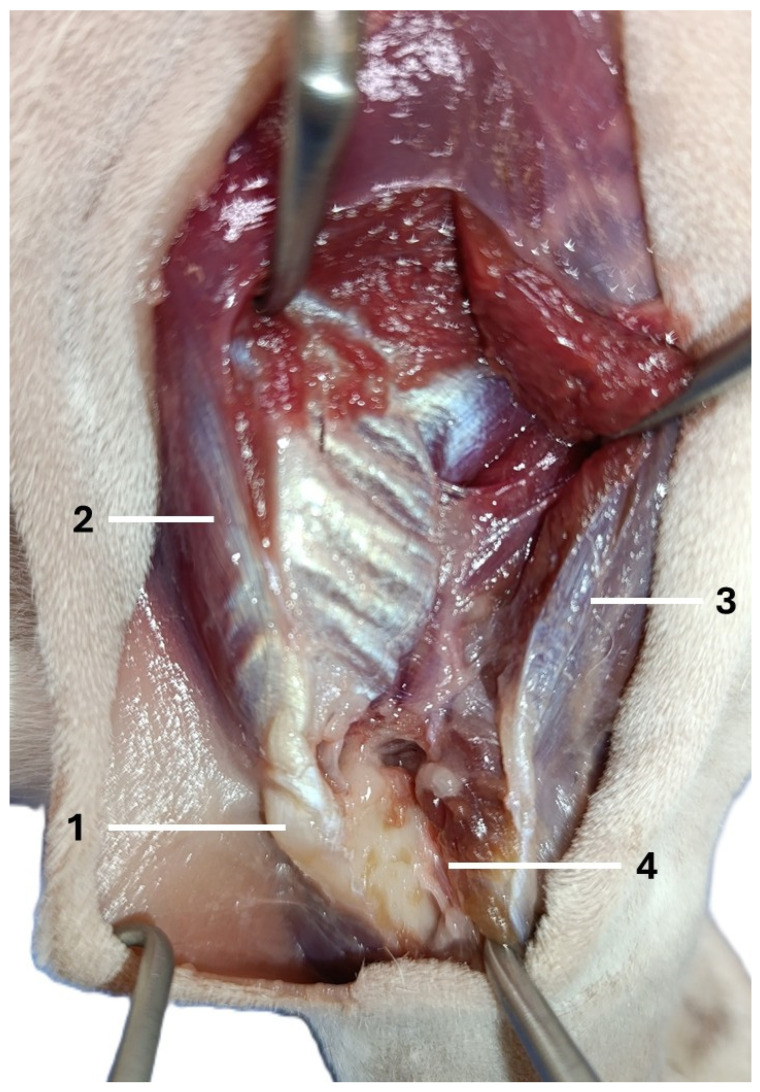
The second step in performing the caudo-lateral approach to the distal humerus (right forelimb): 1—olecranon, 2—long head of the triceps brachii, 3—lateral head of the triceps brachii, 4—anconeus muscle.

**Figure 4:**
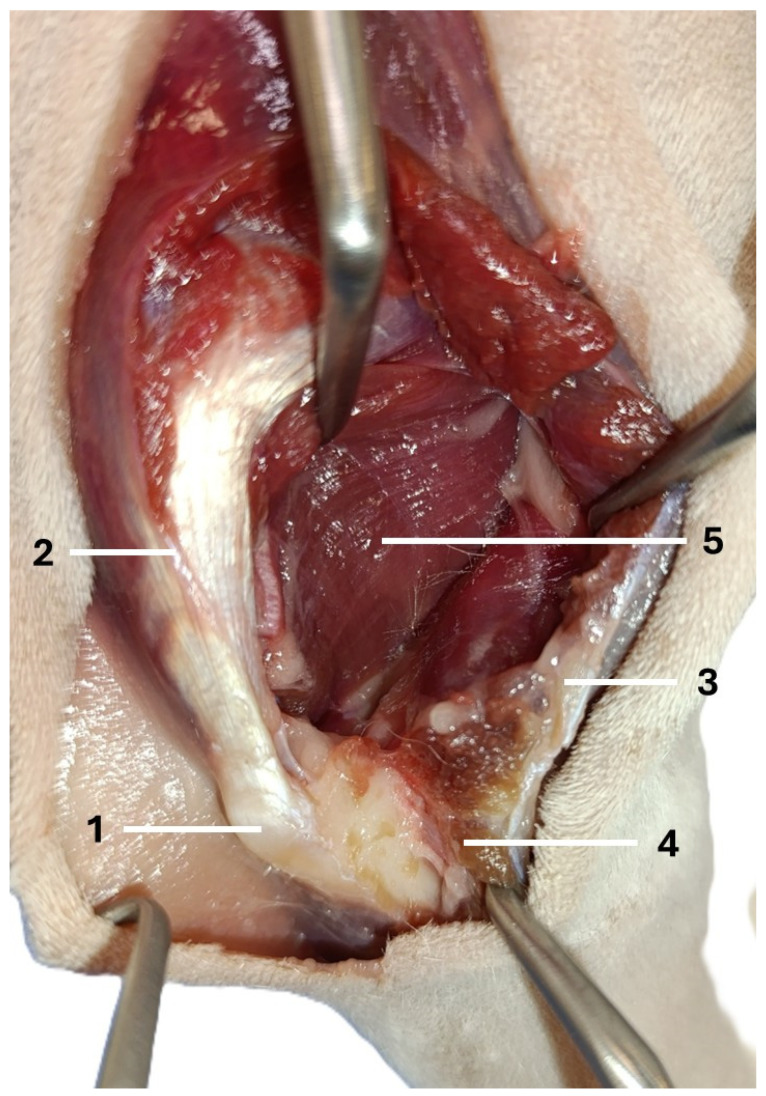
The next step in performing the caudo-lateral approach to the distal humerus (right forelimb): 1—olecranon, 2—long head of the triceps brachii, 3—lateral head of the triceps brachii, 4—anconeus muscle, 5—accessory head of the triceps brachii.

**Figure 5:**
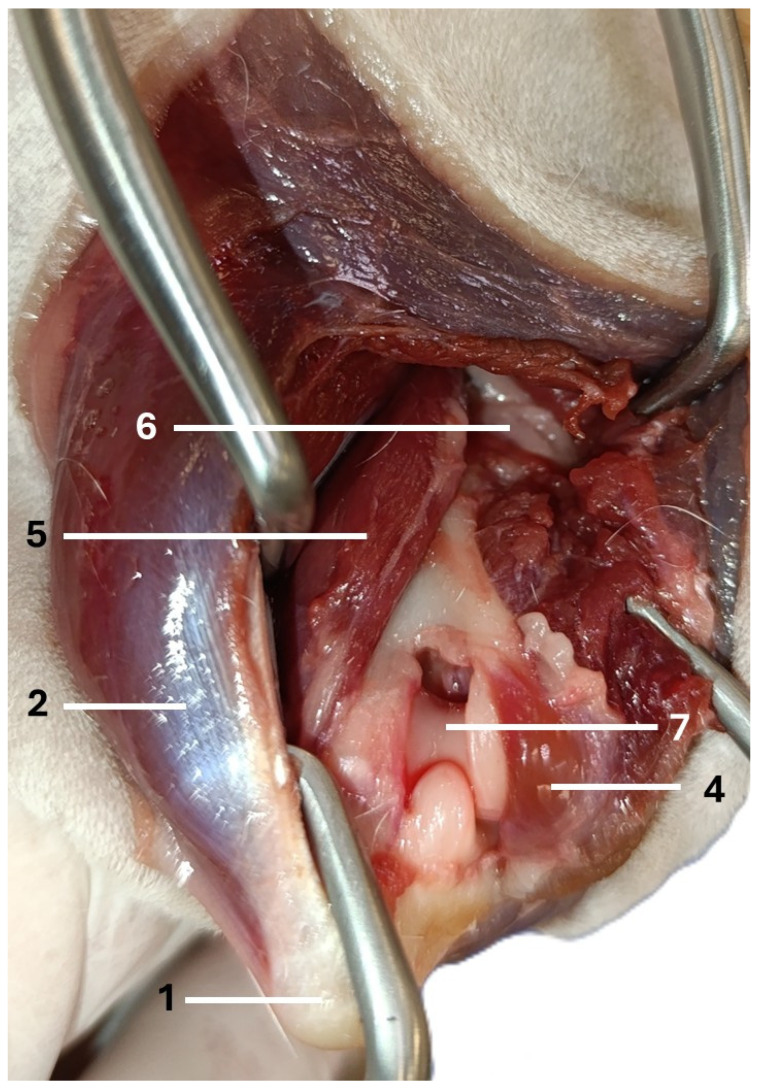
The final step in performing the caudo-lateral approach to the distal humerus (right forelimb): 1—olecranon, 2—long head of the triceps brachii, 4—anconeus muscle, 5—accessory head of the triceps brachii, 6—the radial nerve, 7—humeral condyle.

**Figure 6:**
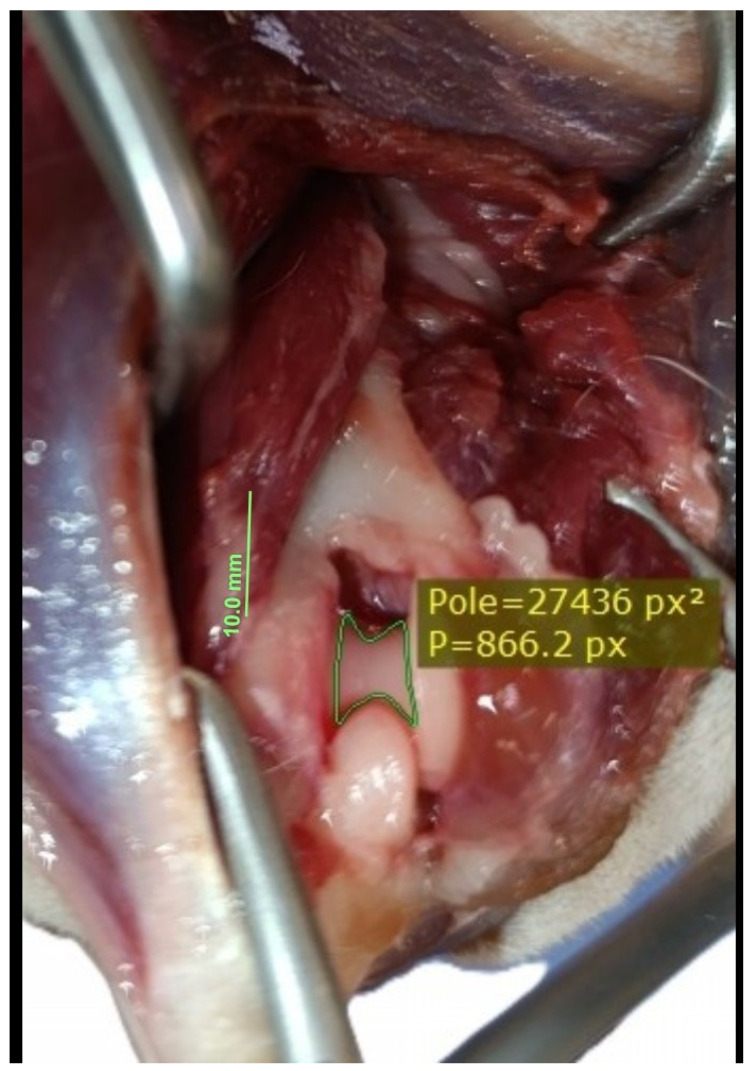
Measurement taken prior to olecranon osteotomy. After delineating the visible margins of the articular cartilage, the software automatically calculated the cartilage surface area in px^2^.

**Figure 7:**
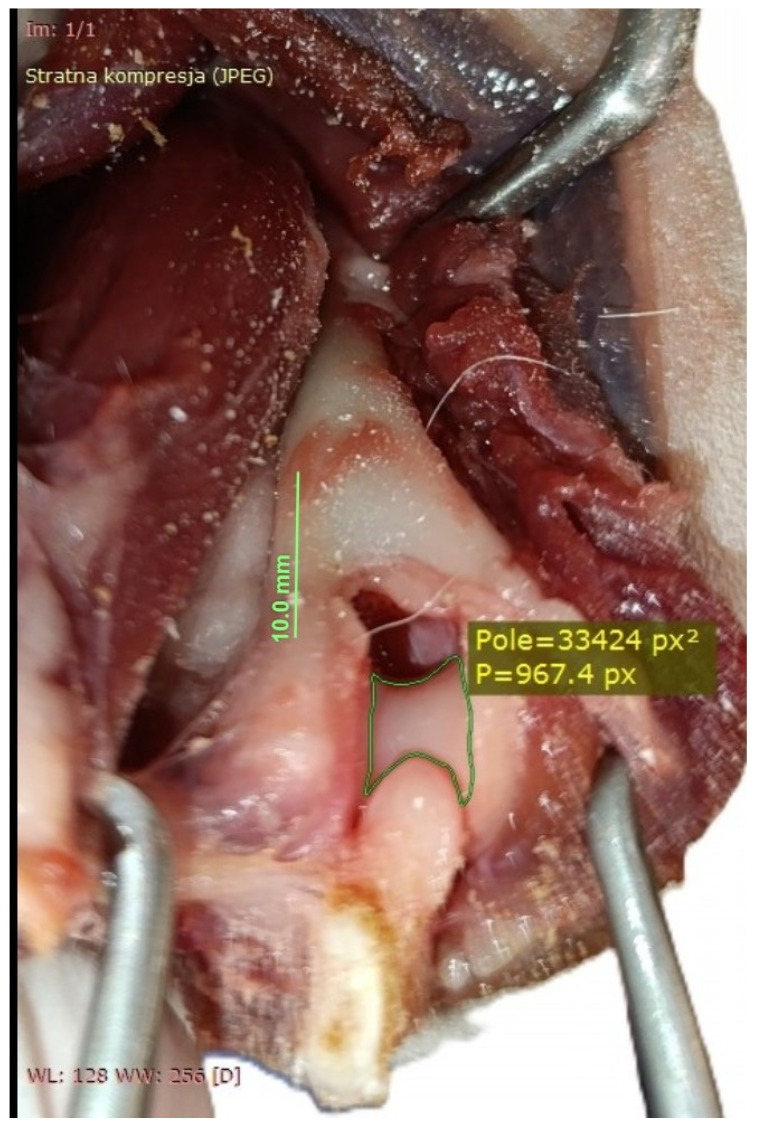
Measurement taken after the olecranon osteotomy. After delineating the visible margins of the articular cartilage, the software automatically calculated the cartilage surface area in px^2^.

The dogs were placed in lateral recumbency, but could be turned into dorsal recumbency if necessary. Their affected limbs were positioned upwards. A caudolateral incision was made. First, the anconeal muscle was cranially and proximally detached from its insertion to the proximal ulna, and a capsulotomy was made. The approach then continued from the distal to the proximal direction between the long and lateral heads of the triceps brachii. Following exposure, the accessory head was separated from the lateral head in a distal-to-proximal direction. Dissection was continued until the humeral shaft and radial nerve were exposed. The radial nerve was identified as a white structure that emerged between the muscle bellies (Figure 2, Figure 3, Figure 4 and Figure 5). After debriding the olecranon fossa and elevating the origin of the anconeus muscle, the articular surface of the humerus was photographed from a distance of 90 mm (as measured by caliper) from the surgeon’s perspective, with the elbow in full flexion. An olecranon osteotomy was then performed, and the visible articular surface was photographed again. The photos were taken with the same smartphone camera at the same resolution (Xiaomi 12 Lite, Beijing, China—main camera: triple lenses, 108 MP, f/1.9, 26 mm (wide), 1/1.52″, 0.7 µm, PDAF, 8 MP, f/2.2, 120° (ultrawide), 1/4.0″, 1.12 µm, 2 MP, f/2.4 (macro)). The photos were calibrated and imported into RadiAnt (Medixant, Poznań, Poland), a program designed to process graphic images. The visible cartilage margins were delineated by the mouse, and then the software automatically calculated the surface area of the cartilage in px^2^, before (A_0_) and after (A_1_) olecranon osteotomy (Figure 6 and Figure 7). The visible area of the articular surface of the humeral condyle after elevation of the anconeus muscle and triceps splitting was calculated as the ratio of the surface area before olecranon osteotomy to the surface area after olecranon osteotomy (A_0_/A_1_) and was expressed as a percentage.

### Statistical Methods

Numerical variables (visible surface areas in px^2^ and their ratio) were examined for normality of distribution using the normal probability Q-Q plots and the Shapiro–Wilk test. Extreme values (outliers) were identified according to Tukey’s method. However, outliers were not excluded from hypothesis testing. Given a significant violation of the normality assumption, all numerical variables were summarized as the median, interquartile range (IQR), and range. The visible surface areas and their ratios were compared between sides using the Wilcoxon signed-rank test and between males and females using the Mann–Whitney U test. Median differences between sides with 95% confidence intervals (CI 95%) calculated according to the exact method [29] were presented. The correlation between dogs’ body weight and the visible areas was examined using Spearman’s rank correlation coefficient (R_s_) with CI 95% calculated according to the method based on the Fisher z-transformation [29]. All tests were two-sided, and the significance level (α) was set at 0.05. Statistical analysis was performed in TIBCO Statistical 13.3 (TIBCO Software Inc., Palo Alto, CA, USA).

## 3. Results

Access to the distal and middle parts of the humerus was achieved in all cadavers. The anatomical landmarks and structures (the olecranon, the anconeus muscle, the long head, the lateral head, and the accessory head of the triceps, the radial nerve, and the distal humerus) were easily identifiable. The only vital structure that could be harmed in this approach was the radial nerve. However, it was easily identified in all dogs. Neither the A_0_ nor the A_1_ differed significantly between left and right elbows (*p* = 0.698 and *p* = 0.605, respectively; Table 1). Therefore, average values from both elbows were used in further analyses. Both A_0_ and A_1_ were significantly positively correlated with dogs’ body weight (*p* < 0.001), and both correlations were strong (R_s_ = 0.92, CI 95%: 0.79, 0.97 and R_s_ = 0.86, CI 95%: 0.63, 0.95, respectively). A_0_ and A_1_ did not differ significantly between sexes (*p* = 0.999 and *p* = 0.751, respectively).

**Figure 8:**
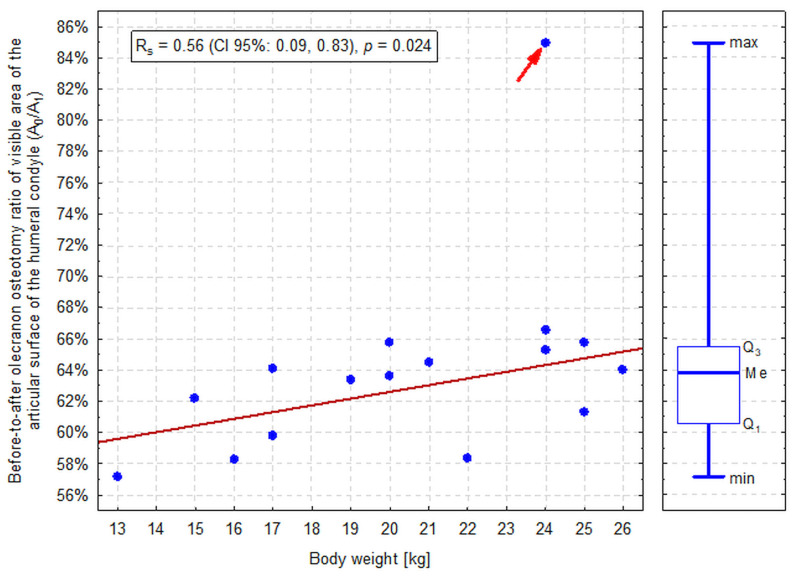
The correlation between the dogs’ body weight and the ratio of the visible area of the articular surface of the humeral condyle before olecranon osteotomy (after elevation of the anconeus muscle and triceps splitting; A_0_) to the visible area after olecranon osteotomy (A_1_). An extremely high value (outlier) is indicated by an arrow. The red line presents a linear trend for 15 observations (excluding an outlier). Median (Me), lower and upper quartile (Q_1_ and Q_3_, respectively) and minimum (min) to maximum (max) range of the A_0_/A_1_ ratio is presented along the vertical axis. R_s_—Spearman’s rank correlation coefficient; CI 95%—95% confidence interval.

Obviously, A_0_ was significantly smaller than A_1_ in all dogs (*p* < 0.001; Table 1). A_0_/A_1_ ranged from 57% to 67% in 15 dogs (median: 64%, IQR: 61%–66%) and in one dog a very high value of 85% was observed (an outlier according to the Tukey’s method; Figure 7). This value resulted from normal A_0_ (39.6 × 10^3^ px^2^) at an abnormally low A_1_ (46.3 × 10^3^ px^2^) for a 24 kg dog (other 2 dogs weighing 24 kg had A_1_ of 61–62 × 10^3^ px^2^). A_0_/A_1_ was significantly positively correlated with the body weight (*p* = 0.024); however, the correlation was weaker than in the case of A_0_ and A_1_ individually (R_s_ = 0.56, CI 95%: 0.09, 0.83; Figure 8). A_0_/A_1_ did not differ significantly between sexes (*p* = 0.290).

## 4. Discussion

We demonstrated that the distal articular surface of the humerus, the epicondyles, and the shaft of the bone could be exposed in dogs using the caudolateral approach. Elevating the anconeus muscle and splitting the triceps provided good access to these structures in all cadavers. During dissection, we identified the radial nerve as the only vital structure. It could then be easily protected. Protecting vital structures, such as nerves, is a crucial aspect of orthopedic surgery. The radial nerve, for example, is essential for the function of the forelimb. However, due to the nature of this study, factors that influence the identification of the radial nerve during surgery, such as post-traumatic oedema or hematoma, were not examined. According to a cadaveric study, smaller vessels accompanying the radial nerve, such as the collateral radial artery and vein, were also not identified. As expected, osteotomy of the olecranon significantly widened access to the condyle. In humans, a cadaveric study confirmed that the transolecranon approach provides greater exposure of the articular surface than the triceps splitting and reflection approach [17]. In our study, the significant widening of the visible area of the articular surface of the humeral condyle (57% to 67%) was observed. However, like the authors of the human study [17], we also believe that such visibility is sufficient, and an olecranon osteotomy will not be necessary in clinical practice after triceps splitting. We also observed that, although the visible part of the area tends to increase in proportion to the dog’s body weight, the overall variation in visibility appears to be low enough to ensure successful access to operated structures, regardless of the dog’s size. It can be assumed that the increase in visible area of articular cartilage depended on the increase in humeral bone size in larger dogs. However, the size of the humeral bone of the examined cadavers was not estimated in this study.

Calculating surface areas is a mathematical process that requires remembering several formulas, all of which are specific to regular shapes. In the case of an irregular shape, the task is more tedious as no direct formula can be applied to it [30]. Nowadays, digital image processing techniques can be used to estimate the surface area of irregular objects [31]. Despite the limitations of photography in relation to the body’s natural curvature, Bhedi et al. [32] compared the results of measuring wound surface area in humans, using photographs, with those obtained by the direct method. In the latter method, sheets of transparency (thin polyester film) were placed directly over the wound, and its perimeter was traced with a permanent marker. The authors stated that photographic and transparency methods provided measurements of the wound surface area with equivalent results. In our study, calibrated digital images were used to calculate the visible area of the articular surface in px^2^. While pixel counts may not accurately represent the true 3D surface area of the humeral condyle, they did provide insight into the differences in the visible area of the articular surface before and after olecranon osteotomy. As the author (P.T.) created both the pictures and the calculations, inter- and intra-observer repeatability were not assessed. This topic warrants further exploration in future studies.

The surgical approach to the bones and joints is always devastating to the surrounding soft tissue. The need for cutting, dividing, and retracting soft tissue causes pathological changes. A good approach to fracture management should enable easy access to the fracture site, preserve all nerves, and prevent unnecessary injury to muscles and the vascular supply [13]. In humans, the transmuscular approach, which involves splitting the muscle along its fibers to provide direct access to the area of interest, is commonly used because it eliminates the need for extensive muscle dissection [16,23,33]. This technique enables access by separating muscle groups and fascicles, allowing the development of intermuscular working channels while preserving muscle integrity and function [34]. Furthermore, it is believed that muscle splitting results in less muscle damage than detaching and retracting the muscle bellies [35]. Similar observations have also been reported in dogs [36]. This study employed a combination of several approaches. Some of these approaches are well known in canine surgery. For example, elevating the anconeus muscle from the proximal ulna facilitates access to the elbow joint and is often used to treat an ununited anconeal process [37]. A broader approach to the anconeal process, e.g., for stabilization, is possible after dissection between the long and lateral heads of the triceps brachii [6,38]. The origin of the anconeus muscle can also be elevated to provide access to the subcondylar region. This technique is used in the transolecranon approach to treat T-Y fractures [7,10]. The anconeus muscle in dogs helps keep the extended elbow in the stance phase [39]. This muscle originates from the caudal aspect of the distal humerus. It runs laterally along the lateral supracondylar crest to the lateral epicondyle and medially to the medial epicondyle, occupying the olecranon fossa of the humerus. Finally, it continues laterally over the elbow joint capsule and inserts into the proximal ulna. The veterinary literature contains no information on complications arising from the surgical elevation of the anconeus muscle. Nevertheless, it appears that this muscle plays a vital role in the movement of the elbow joint in certain dog breeds. Generally, the canine anconeus muscle is composed of type I (slow-twitch) fibers, whose main function is to resist elbow flexion when the dog is standing. However, in greyhounds, 49% of the anconeus muscle is composed of type IIa fibers (fast-twitch and fatigue-resistant) [40]. This suggests that this muscle plays a significant role in locomotion by actively extending the elbow in this breed. Shaff et al. [39] identified differences in the structure and function of the anconeus muscle as the reason for lameness in young greyhound following injury to this muscle. As the function of the anconeus muscle in dogs is unclear (whether it only stabilizes the elbow or actively extends it), its continuity should be restored, and the insertion sutured to the fascia and periosteum of the lateral epicondyle and proximal ulna after surgery.

The second muscle dissected in this study was the triceps brachii. This muscle plays a crucial role in forelimb movement in dogs and is characterized by its anatomical structure, which consists of four heads [41]. The medial head has a small muscle belly and terminates on the medial aspect of the olecranon. The other heads of the triceps brachii muscle have a blended tendon insertion, comprising the long head, lateral head, and accessory head [27]. In this experiment, the triceps tendon was divided. The dissection continued between the long and lateral heads, and then between the lateral and accessory heads. Such an approach in clinical cases necessitates triceps tendon repair at the end of the surgical procedure [42]. Osteotendinous disruption necessitates a tendon–bone suture through bone tunnels [43,44].

For comparison to the presented approach, which gives good exposure of the distal and middle humerus, the latero-medial approach appears to be more extensive, requiring the surgeon to perform a wide dissection between the muscle fibers, e.g., the superficial pectoral muscle, between the muscle bellies, and retract large muscles, such as the biceps brachii or brachiocephalicus. Additionally, the radial nerve, median nerve, ulnar nerve, brachial artery, and vein must also be identified and protected [4,5]. Despite such wide access, the accuracy of articular reduction is usually assessed indirectly by checking the alignment of the lateral and medial epicondyles, because the visibility of the articular surface is limited [5,7]. Fluoroscopy and/or elbow arthroscopy can be employed to improve the accuracy of articular surface reconstruction [3]. However, this necessitates specialized equipment and skills.

This study has some limitations. The experiment was conducted on fresh-frozen cadavers, thawed before preparation. The use of live animals for experimental and educational purposes has decreased significantly in response to public concerns, resulting in cadavers becoming a more common resource for practical training [45]. Although formaldehyde-preserved cadavers are suitable for learning anatomy, they lack the characteristics of live patients necessary for surgical training, particularly for procedures requiring good tissue flexibility [46,47]. Ideally, we would use fresh cadavers for the experiment, but we lack the resources to conduct a separate experiment on each one. This is why we used a cohort of fresh-frozen cadavers. Such cadavers are still a good material for training in surgical procedures, as they offer lifelike anatomical conditions and realistic tissue handling [48]. The cadavers used in this experiment exhibited good soft tissue flexibility following thawing, enabling an adequate surgical approach, but the absence of intraoperative bleeding is a strong limitation of this study. A second limitation is that the cadavers used had intact elbows. T-Y fractures are difficult to manage. The proposed approach should therefore be tested on cadavers with such fractures, as well as in clinical cases where soft tissue oedema and muscle contracture can be observed. In certain clinical scenarios where screwing the distal part of the medial plate is performed without an olecranon osteotomy, an additional medial paratricipital approach may facilitate the procedure [48,49].

## 5. Conclusions

The proposed approach provides good exposure of the humeral condyle, the supracondylar region, and the diaphysis. Good exposure of such structures can be particularly useful when treating complex fractures of the distal humerus in heavy, active patients where robust fracture fixation is required. Further studies are needed to evaluate the ease and effectiveness of reduction and instrumentation for T-Y fractures using the caudolateral approach to the humerus. The efficiency of the extensor mechanism after triceps splitting also needs to be evaluated.

## Figures and Tables

**Table 1:** The visible area of the articular surface of the humeral condyle after elevation of the anconeus muscle and triceps splitting expressed as the median, interquartile range (IQR), and range.

Visible Area	Left Elbow (*n* = 16)	Right Elbow (*n* = 16)	Median Difference (CI 95%)	*p*-Value ^a^
Before olecranon osteotomy (A_0_) [×10^3^ px^2^]	38.7, 25.0–40.3 (16.5–41.4)	38.0, 27.1–39.6 (17.0–41.0)	0.36 (−0.65, 0.76)	0.698
After olecranon osteotomy (A_1_) [×10^3^ px^2^]	59.2, 41.9–61.5 (29.6–66.2)	59.1, 41.4–61.1 (29.0–66.0)	0.14 (−0.50, 0.67)	0.605
A_0_ to A_1_ ratio (A_0_/A_1_)	63.3%, 58.7–64.9% (55.6–85.8%)	63.6%, 60.1–65.4% (58.1–84.1%)	0.3% (−3.0%, 1.4%)	0.877

^a^ Wilcoxon signed rank test.

## Data Availability

The data presented in this study are available in the article.
